# Laparoscopic re-sleeve gastrectomy for weight regain after modified laparoscopic sleeve gastrectomy: first case report and surgery in South America

**DOI:** 10.1590/0102-6720201600S10033

**Published:** 2016

**Authors:** Eduardo Henrique PIROLLA, Felipe Piccarone Gonçalves RIBEIRO, Fernanda Junqueira Cesar PIROLLA

**Affiliations:** Harvard Medical School, Boston, Massachusetts, USA

**Keywords:** Gastrectomy, Weight gain

## INTRODUCTION

Modified laparoscopic sleeve gastrectomy (MLSG) is a great option to control diabetes mellitus type II, obesity and other co-morbidities. However, a common challenge of bariatric surgeries is weight regain in the long term. 

Re-sleeve gastrectomy started a few years ago and is suggested to be a feasible option to manage these situations. In Latin America there is no case reported of re-sleeve gastrectomy after MLSG at now.

Therefore we present a case report of an individual submitted to re-sleeve gastrectomy after weight regain after seven years of MLSG.

## CASE REPORT

LSF, 48 year old caucasian, male, Brazilian went to private practice in São Paulo, in 2009, presenting the following conditions: severe obesity with BMI of 47, type II diabetes mellitus, hepatic steatosis and hypertension. Therefore, he had a metabolic syndrome and a severe obesity. He was submitted to the following exams: peptide C (2.86), anti-GAD antibody and anti- insulin antibody negative and fasting glucose (285 mg/dl) with Januvia and Glifage usage.

**FIGURE 1:**
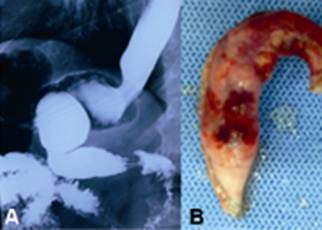
A) Contrasted esophagus, stomach and duodenum demonstrating moderate fundus dilatation; B) surgical specimen of re-sleeve (12 cm of gastric fundus)

MLSG (Figure 1) was performed in 2009. It basically consists in the removal of part of the gastric fundus and body of the stomach up to one inch from the pylorus vein, reducing the production of ghrelin^4^. In the following eight months after surgery, patient moved from BMI of 47 to 27.5. As a result he got his diabetes and metabolic syndrome controlled. Seven years later returned referring the capacity of eating a larger volume and weight regain. His new BMI was 34,5. Given this clinical scenario were requested abdominal ultrasound, oral contrasted esophagus, stomach and duodenum and upper gastrointestinal endoscopy (Figure 1A).

Laparoscopic cholecystectomy with cholangiography was performed and also a partial gastric fundus re-sleeve (Figure 1B) was executed using articulated linear stapler and load-blue clips and reinforcement over suture with polidioxanone 3-0. Surgery obtain great results and without any intraoperative and postperative complications. Patient stayed in hospital for 48 h. 

After six months of the procedure he had no complication, 12 kg weight loss and stopped all medications. He presented a change in BMI=8%, excess BMI loss (%EBMIL) of 84.21% and percent of total weight loss (%TWL) of 12.37%.

## DISCUSSION

Literature present few publications describing re-sleeve gastrectomy. None of them in the Latin-America and none reporting MLSG as the primary bariatric procedure.

In 2006, Baltasar A, et al. reported two patients that were submitted to laparoscopic sleeve gastrectomy and when they regained weight, laparoscopic re-sleeve gastrectomy and duodenal switch were performed and reduced patients BMI after 3-4 months^1^. However, duodenal switch is a best indication for a super-super-obesity and a very malabsorptive technique. Re-sleeve is a good way to approach cases which patient´s need to loss the great part of weight which re-gained without other problems.

In 2009, Iannelli A, et al. performed a feasibility study of revision of laparoscopic sleeve gastrectomy. They recruited 13 patients with weight regain or insufficient weight loss. They followed their patients in the 1^st^, 6^th^ and 12^th^ months after revision in laparoscopic sleeve gastrectomy. Before surgery the mean BMI was 44.6 kg/m^2^; one month after surgery the mean BMI was 32.3 kg/m^2^; six months after surgery the mean BMI was 32 kg/m^2^ and 12 months mean BMI was 27.5 kg/m^2^. They concluded that for one year after revision of laparoscopic sleeve gastrectomy the procedure was safe and effective^3^.

Rebibo L et al. compared repeat sleeve gastrectomy with primary sleeve gastrectomy. They found that repeated sleeve gastrectomy can generate similar weight loss then primary sleeve, but can be associated with an increased risk of complications, such as gastric fistula^5^.

In 2014 Cesana G et al. reported their results showing 201 patients that were submitted to re-sleeve gastrectomy. They reported no intra and postoperative complications and also a reduction of antihypertensive and hypoglicemic drugs in patients with diabetes and hypertension after re-sleeve procedure^2^.

In short term safety, our results are consistent with literature since no pre or postoperative complication occurred. Our results are also similar to Cesana according to the reduction of the number of hypoglicemic agents. We must continue following this patient to check if results are consistent in middle and long term.

Our main limitation was our sample size of only one patient. To have more solid results larger studies are necessary.
